# A randomized controlled trial protocol to test the efficacy of a dual-task multicomponent exercise program in the attenuation of frailty in long-term nursing home residents: Aging-ON_DUAL-TASK_ study

**DOI:** 10.1186/s12877-018-1020-z

**Published:** 2019-01-08

**Authors:** Chloe Rezola-Pardo, Haritz Arrieta, Susana Maria Gil, Jose Javier Yanguas, Miren Iturburu, Jon Irazusta, Begoña Sanz, Ana Rodriguez-Larrad

**Affiliations:** 1 Department of Physiology, Faculty of Medicine and Nursing, University of the Basque Country (UPV/EHU), Barrio Sarriena s/n, E-48940 Leioa, Bizkaia Spain; 2 Fundación Bancaria “La Caixa”, Plaça Weyler, 3, 07001 Palma, Illes Balears Spain; 3 Matia Instituto, Camino de los Pinos 35, E-20018 Donostia-San Sebastian, Gipuzkoa Spain; 4 BioCruces Health Research Institute, Plaza de Cruces, 48903 Barakaldo, Bizkaia Spain

**Keywords:** Dual-task, Frailty, Exercise, Physical activity, Long-term nursing home, Cognitive frailty, Dual-task intervention, Older adults, Aging

## Abstract

**Background:**

The purpose of the Aging-ON_DUAL-TASK_ study is to determine if a supervised dual-task program carried out in long-term nursing homes is able to attenuate frailty in a greater extent than the same multicomponent exercise program alone.

**Methods:**

This multicenter randomized controlled trial will include 188 participants who will be randomly allocated to either a multicomponent exercise program or to the same multicomponent program with simultaneous cognitive training (dual-task training). Inclusion criteria are as follows: ≥ 70 years, ≥ 50 on the Barthel Index, ≥ 20 on the Mini Examen Cognoscitivo (MEC-35) who are able to stand up and walk independently for 10 m. Subjects in the multicomponent group will attend a twice-a-week multicomponent exercise program of 1-h duration per session, consisting of strength and balance exercises. Participants in the dual-task group will perform the same multicomponent exercise program with concurrent individually tailored cognitive tasks. Study assessments will be conducted at baseline and at 3 months. The primary outcome measure will be gait speed under dual-task conditions and secondary outcomes will include physical fitness measurements, gait spatiotemporal parameters, cognition and emotional assessments, several frailty scales and objectively measured physical activity.

**Discussion:**

The present research will add valuable information to the knowledge around the effects of the dual-task program in long-term nursing home residents, taking altogether physical, cognitive and emotional variables linked to frailty.

**Trial registration:**

Australian and New Zealand Clinical Trials Registry (ANZCTR) with the identifier: ACTRN12618000536268. Registration date: 11/04/2018.

## Introduction

The global increase in life expectancy and consequent aging of the population, leads to estimates that the number of dependent older adults will rise from 350 million in 2010 to 488 million by 2030 [1]. Accordingly, the number of older adults living in long-term nursing homes will also increase considerably. Older adults in long-term nursing homes represent a complex and heterogeneous population with a high prevalence of dependence in the activities of daily living, cognitive impairment, depression, high fall rates, multimorbidity and polymedication [2, 3]. In addition, long-term nursing home residents tend to be extremely inactive, engaging in sedentary activities for most of the day [4]. Thus, providing the best care for this population has become a challenge for both social and health care services [5].

In the last few years, research in aging has focused on frailty syndrome. Frailty is considered a state of vulnerability highly prevalent among the older adult population [6–8]. Although frailty has traditionally been described as a purely physical syndrome, a number of epidemiological studies have reported that frailty increases the risk of future cognitive decline and that cognitive impairment increases the risk of frailty, suggesting that physical frailty and cognitive impairment interact [9]. Cognition declines with age, with normal subtle cognitive changes that may affect everyday life functioning [10] and frail older adults usually perform worse in certain executive function and processing speed tests [11]. Recently, the International Academy on Nutrition and Aging (I.A.N.A) and the International Association of Gerontology and Geriatrics organized an International Consensus Group on “Cognitive Frailty”, which proposed a definition of cognitive frailty [12] and suggested that all frail older adults should undertake a complete cognitive evaluation, including executive function tests [13].

In consonance with this idea, an impaired capacity to perform attention-demanding mobility activities such as carrying out two tasks (physical + cognitive) simultaneously, also known as *dual-task*, could be a novel marker of physical and cognitive frailty. Many activities of daily life involve the performance of several tasks at a time, creating competing demands for attentional resources that challenge both motor and cognitive functions [14]. Considering that attentional capacity is limited, when demands exceed capacity, performance of dual-tasks can be affected compared to performance of the same tasks in a *single-task* fashion. Indeed, older adults show greater impairments compared to their younger counterparts in cognitive-motor dual-task performance, such as naming animals while walking or making calculations during balance exercises [15, 16]. Additionally, low dual-task performance capacity is associated with cognitive impairment and with a high risk of falling [17–19].

Previous studies of dual-task as a test of functional performance in older adults have focused on the gait speed test or on the Timed Up and Go test as physical tasks, while introducing semantic fluency or a calculus command as cognitive content. The difference between performance in the dual-task and the single-task tests is known as *dual-task interference* or *dual-task cost* [20]; this difference increases with aging [21]. The dual-task cost can be accounted for in both the physical and the cognitive domains. In this regard, the most commonly used formula is: dual-task cost = ((dual task − single task) single-task) × 100% [18]. For the physical dual-task cost calculation, time in seconds is used, whereas for the cognitive dual-task cost, either the number of correct responses or the percent of correct responses can be used to compare between single and dual task performances.

Dual-task performance can be modified with specific dual-task training [22, 23]. In fact, this type of intervention can maintain or even improve cognitive function [24, 25], especially executive function [26]. Two models have been suggested that might explain training-mediated dual-task performance changes [27]. The task-automatization model is based on the assumption that individual tasks can be automatized and predicts similar improvements either with single-task or dual-task training [27]. Alternatively, the task-integration model advocates for the efficient integration of both tasks through dual-task training, resulting in dual-task performance improvements [27]. According to the latter model, dual-task performance would only improve after dual-task but not single-task training. Furthermore, dual-task training may be superior to single-task training [28–30], since dual-task training requires greater cognitive and motor resources and is more complex in terms of control and coordination demands. Pellecchia et al. [31] observed greater improvements in postural sway under dual-task conditions after dual-task training compared to single-task training, supporting the task-integration model and suggesting that both physical and cognitive functions interact in a way still to be revealed.

To our knowledge, no studies have explored the effects of a supervised dual-task multicomponent exercise intervention in long-term nursing home facilities from a broad perspective of frailty, assessing functional capacity under single-task and dual-task conditions, physical activity, cognitive performance and emotional status. Therefore, we designed a randomized multicenter study, the Aging-ON_DUAL-TASK_ study, to test hypothesis that the addition of cognitive training to a supervised multicomponent exercise program can improve gait speed performance under dual-task conditions by, at least 0.08 m/seg in a population of older adults in long-term nursing homes. The major aim of the Aging-ON_DUAL-TASK_ study is to determine if a supervised dual-task program carried out in long-term nursing homes can attenuate frailty to a greater extent than the same multicomponent exercise program without cognitive training.

The Aging-ON_DUAL-TASK_ study is based on a previous study [32, 33] in which feasibility regarding recruitment, adherence and safety of the multicomponent exercise program were successfully ascertained. A pilot study was performed to refine the outcome assessments, establish the progression of the cognitive training, and optimize the organizational infrastructure.

## Methods

### Study design and participants

**Fig. 1:**
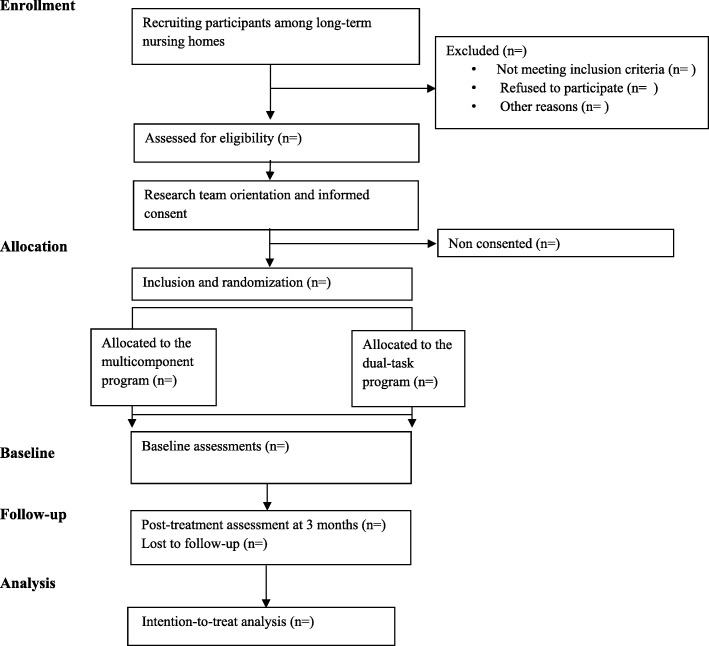
Flow diagram of the study

Based on the proposed objective, an experimental multicentre simple randomized study was designed (Aging-ON_DUAL-TASK_). Participants will be randomly allocated to either a multicomponent exercise program or to the same multicomponent program with simultaneous cognitive training (dual-task program). Participants will be recruited from eight long-term nursing homes in Gipuzkoa, Basque Country, Spain. Each site will enroll a minimum of 16 subjects and interventions will take place between June 2018 and December 2018. Researchers responsible for data gathering will be blinded to group assignment. The assessments will be carried out by research staff at baseline and at 3 months after the beginning of the intervention. The study has been designed and results will be reported following the CONSORT Statement extension for trials of non-pharmacological interventions and pragmatic intervention trials (Fig. 1).

### Inclusion and exclusion criteria, recruitment, and randomization

The inclusion criteria, recruitment, and randomization methodologies in the Aging-ON_DUAL-TASK_ study will be the same as on a previously published protocol [33]. The inclusion criteria will be: age ≥ 70 years old; a Barthel Index [34] score ≥ 50 and score ≥ 20 on the MEC-35 Test [35] [Mini-examen cognoscitivo, an adapted and validated version of Mini Mental State Examination (MMSE) in Spanish]; and capacity to stand up and walk independently for at least 10 m. Participants will not be eligible if they are judged clinically unstable by the medical staff, or have in any other condition in which entering the study would not be in the subject’s best interests.

Identification of individuals that meet the inclusion criteria will be facilitated by the databases of the included long-term nursing homes. The primary recruitment strategy will be information provided to the potential participants by the medical and nursing professionals from each facility. All volunteers will receive detailed study information at their reference sites through the research team: objectives, measurement variables, and other details about the interventions will be explained orally and in writing to both potential participants and their families. After signing the informed consent, the participants within each center will randomly be assigned (in a 1:1 ratio) through sealed opaque envelopes to either the multicomponent or the dual-task group by coin-tossing sequence generation.

### Multicomponent exercise program

Feasibility and safety of the multicomponent exercise program were ascertained in a previous study which included full details on volume, intensity, and type of strength and balance exercises [32, 33]. Participants allocated to this group will attend a twice-a-week multicomponent exercise program of one-hour duration per session, consisting of strength and balance exercises conducted by an experienced physical trainer. Participants will also continue attending their usual activities and workshops.

### Dual-task program

In the dual-task program, individually tailored cognitive tasks relying predominantly on executive function will be conducted concurrently with approximately four of the multicomponent exercises (Table 1).

**Table 1:** Programation of the intervention for the 5th week

Objective	Sesion 1	Sesion 2
Warm-up 5 min	Range of motion for different joints	Range of motion for different joints
Strength training	–	Arm curl 60% 2 sets 8–12 rep + DAT (cog)
	Chair stand 60% 2 sets 8–12 rep	Chair stand 60% 2 sets 8–12 rep
	Leg flexion 60% 2 sets 8–12 rep + DAT (cog)	–
	–	Leg extension 60% 2 sets 8–12 rep + Inhibition task
	Leg abduction 60% 2 sets 8–12 rep + Calculus task	–
	–	Hip extensión 60% 2 sets 8–12 rep
	Standing on tips and heels 3 sets 10 rep + SAT	Standing on tips and heels 3 sets 10 rep + SAT
Balance training	–	Feet together stance 2 sets 10 s + DAT (physical)
	One legged stand 2 sets 10 s	–
	Semi-tandem/Tandem 2 sets 10 s + DAT (physical)	Semi-tandem/Tandem 2 sets 10 s + DAT (cog)
	Circuit training 2 sets	–
	–	Stepping 2 sets 10 rep
	Ball reaching 2 sets + Semantic memory	–
Cool down 5 min	Stretching, breathing, relaxing exercises.	Stretching, breathing, relaxing exercises.

*rep* repetitions, *DAT* divided attention task, *cog* cognitive, *SAT* sustained attention task

The challenge of dual-task-s will be increased by augmenting the complexity of motor tasks (progressing from sitting to standing and from static to dynamic exercises, reducing base of support, etc) (Table 2) and/or cognitive tasks (number of stimuli, complexity of word categories, etc.) (Table 3).

**Table 2:** Detailed description of the general DT group intervention

3 MONTHS			
Objective	1ST MONTH Familiarisation phase	2ND MONTH Strength development Static balance DT	3RD MONTH Strength maintenance Dynamic balance DT
Strength	3–4 ex: 1–2 sets, 8–12 rep at 40–50% of 1RM	4–5 ex: 2 sets, 8–12 rep at 60% of 1RM	4–5 ex:1–2 sets, 8–12 rep at 65–70% of 1RM
Balance	2–3 ex, progressive difficulty in sitting position and progressing to standing position.	4–5 ex, progressive difficulty in standing position with decreasing arm support and increasing instability.	
Dual-task	In 3–4 of strength ex	In 2–3 of strength ex and 1–2 of balance ex	In 1–2 of strength ex and 3–4 of balance ex

*ex* exercises, *rep* repetitions

**Table 3:** Progression of complexity of secondary tasks by levels of difficulty

Main cognitive function		Level 1	Level 2	Level 3	Level 4	Level 5
Attention	Divided (cognitive) *CATEGORIES:Buildings/ Dairy products/ colors*	The participant will repeat a specific word from a certain category (e.g house) every time the instructor says *it*	The participant will raise a hand every time the instructor says a specific word from a certain category or when a green card is presented	The participant will raise a hand every time the instructor says two specific words from certain categories or when a green card is presented	The participant will raise a hand every time the instructor says two specific words from certain categories or when a green card is presented or when the instructor claps	The participant will raise a hand and repeat the word every time the instructor says two specific words from certain categories or when a green card is presented or when the instructor claps
	Divided (physical)	Participants will carry out the physical task whilst maintaining a cup with a ball upright to avoid the ball from falling				
	Sustained	Naming months of the year forward	Naming months of the year forward starting from a random month	Naming even or odd months of the year forward	Naming months of the year backwards	Naming months of the year backwards starting from a random month
	Shifting	Participants will be asked to shift focus from a cognitive task to another on some of the dual tasks				
Semantic fluency		Naming colors/days of the week/names	Naming members of the family/clothes	Naming professions	Naming cooking instruments or general tools	Naming fish, dog or tree types
Inhibition		If the instructor says *YES* they respond *NO* and viceversa	Every time the instructor says HEADS they have to answer TAILS and viceversa and Previous entry	If a green card is presented they have to say RED and when a red card is presented they have to say GREEN	Level 2 and level 3 instructions altogether	If the word RED is presented in a green color they have to say *GREEN* and vice versa and when the word YES in red color or NO in green color, they have to name the colo*r*.
Problem solving (calculus)		Counting by twos starting from a number ≥ 30	Counting by threes from a number ≥ 50	Substracting by twos from a number ≥ 30	Substracting by threes from a number ≥ 50	Substracting by fours from a number ≥ 100
Movement coordination		Inherent to the muticomponent exercise program				
Movement learning and sequencing						

The first week of the intervention will mainly serve to familiarize participants with the strength and balance exercises and adjust the level of difficulty of each cognitive function task to every participant in the group. In the second week, strength tests will be performed to individualize strength training and ensure training intensity. Throughout the following weeks, dual tasking will be applied mostly in strength exercises to train for divided attention allocation and will progressively move to balance exercises to optimize training adaptations and mimic everyday situations that require double tasking and increasing instability.

Cognitive training will be conducted based on six main cognitive functions essential for everyday life activities (Table 3). One of the most important functions to train is attention, which will be applied in form of: 1) divided attention tasks (with a secondary physical or cognitive task) where participants will have to divide their attention to ensure task achievement; 2) sustained attention tasks, in which attention will have to be maintained throughout a certain time period (1–2 min); 3) shifting, where participants will have to shift their focus of attention between cognitive tasks. In addition, semantic fluency will consist of naming words according to different categories with increasing difficulty such as naming animals, professions or even dog breeds. Other executive functions including calculus or inhibitory control will also be trained, the latter consisting of overriding the natural response to certain stimuli. Finally, due to the fact that movement coordination, movement learning and sequencing are inherent to any exercise-based program, these will be present in both the multicomponent exercise program group and in dual-task group.

### Outcome measures

The primary outcome measure will be gait speed under dual-task conditions. The distance to be covered will be 9 m on a smooth non-slippery surface with starting and ending points marked on the floor with tape. The cognitive task to be performed will previously be explained to participants. Straight after the explanation, the participant will be asked to walk at a comfortable pace on a straight line while simultaneously performing the cognitive task. Time to perform the test will be measured following the procedure described by Bohannon [36]. Gait speed will be then calculated dividing the covered distance (in meters) by the employed time (in seconds).

In addition, both gait spatiotemporal parameters (cadence, single and double support time, etc.) and cognition (number of correct, incorrect and total responses) will be analyzed, and compared with performance in the single-task modality. This difference is referred to as dual-task cost, and will be calculated using the formula: ((dual task – single-task)/single-task × 100) [18].

Secondary outcome measures will include functional (Table 4), cognitive, and emotional assessments (Table 5). Functional capacity will be determined by the following tests (Table 4): the Short Physical Performance Battery test [37] (SPPB); the Senior Fitness Test [38] (SFT); the instrumented Timed Up and Go test [39] (iTUG; BTS Biomedical G-WALK triaxial accelerometer and gyroscope); usual walking speed [36]; the handgrip strength test [40] (Jamar dynamometer) and Berg balance test [41]. Frailty assessment will include the Tilburg Frailty index [42], the Frailty index [43] and the Rockwood clinical frailty scale [44]. In addition, participants will wear an accelerometer (Actigraph GT3X model (Actigraph LLC, Pensacola, FL, USA)) on the hip with a belt for 7 days to measure active and sedentary periods during everyday life, by daily step quantification. Active-period intensities will be classified as light, moderate or vigorous based on Freedson and colleagues’ criteria [45] and recorded in minutes.

**Table 4:** Functional assessment tests

Test (Reference)	Functions/Parameters	Description
Short Physical Performance Battery (SPPB) (Guralnik et al., 1994)^a^	Lower extremity function: static balance, gait speed and getting in and out of a chair	Side-by-side, semi-tandem and tandem stands (10 s); 4 m walk test at comfortable speed and 5 quickly sit to stand from a chair without upper extremity assistance
Senior Fitness Test (Rikli & Jones, 2007)^a^	Upper and lower extremity strength and flexibility, static and dynamic balance and aerobic capacity	Chair-stands in 30 s; 6-min walking test; arm curl test (30 s); chair sit and reach; back scratch and 8 Foot Up and Go test
Instrumented Timed Up and Go test (BTS Biomedical G-WALK) (Mathias et al., 1986)^a^	Dynamic balance	Get up from a chair, walk 3 m at a normal pace, turn and walk back to sit down again
Instrumented walking speed (BTS Biomedical G-WALK) (Bohannon et al., 1996)^a^	Standard gait parameters: speed, step frequency, cadence	Walk for 4 and 9 m at comfortable speed
Bilateral handgrip strength test (Jamar dynamometer) (Fess, 1992)^a^	Hand grip strength	Squeeze the dynamometer with maximum isometric effort for about 5s
Berg balance test (Berg et al., 1992)^a^	Postural stability	Performance of 14 functional tasks
Accelerometry [Actigraph GT3X model (Actigraph LLC, Pensacola, FL, USA)] (Freedson et al., 1988)^a^	Active and sedentary periods during everyday life	7 days period quantification of the number of steps performed per day and minutes completed at light, moderate or vigorous intensity

^a^Rodriguez-Larrad et al. [33]

**Table 5:** Cognitive and Emotional assessment tests

Test (Reference)	Functions	Description
Montreal Cognitive Assessment (MoCA) (Coen et al., 2016)^a^	Mild Cognitive Impairment, Early Alzheimer’s disease	Covered domains: attention and concentration, executive functions, memory, language, visuoconstructional skills, conceptual thinking, calculations, orientation
Wechsler Adult Intelligence Scale (WAIS-IV) (Wechsler et al., 2010)	Cognitive impairment	Covered domains: attention, visual scanning, motor speed
Trail Making Test (TMT) (Reitan, 1958)	Cognitive impairment	Assesses: visual-conceptual and visual-motor tracking, sustained attention and task alternation abilities
Rey Auditory Verbal Learning Test (RAVLT) (Lezak, 1995)	Memory and learning capacity	Evaluates short- and long-term verbal memory assessing the ability to learn a list of 15 common words
Anxiety and Depression Goldberg Scale (Goldberg et al., 1988)^a^	Affective state	Includes nine depression and nine anxiety items from the past month
The Jong Gierveld loneliness scale (de Jong-Gierveld, 1987)	Emotional and social loneliness	Includes characteristics of the social network, background variables, personality characteristics, and evaluative aspects
Questionnaire QoL-AD (Logsdon et al., 2002)	Perceived quality of life	Self-rated quality of life for people with cognitive impairments

^a^Rodriguez-Larrad et al. [33]

For cognitive and emotional assessment (Table 5), participants will be assessed through the Montreal Cognitive Assessment [46] (MoCA), the Coding and Symbol Search test (which provide a measure of processing speed) from the Wechsler Adult Intelligence Scale, Fourth Edition (WAIS-IV) [47], the Trail Making Test part A [48] (TMT), the Rey Auditory Verbal Learning Test [49] (RAVLT), the Anxiety and Depression Goldberg Scale [50], the Jong Gierveld loneliness scale [51], and the Quality of Life Alzheimer’s disease scale [52] (QoL-AD).

The following additional variables will also be registered: sociodemographic variables: age, gender, socioeconomic situation, educational level, and marital status; level of independence in activities of daily living: Barthel index [34]; cognitive impairment assessed through MEC-35 [35]; anthropometric measurements: weight, height, body mass index, waist and hip circumferences, and waist-to-hip ratio; and clinical outcomes Charlson comorbidity index [53], number of falls, visits to the emergency service, number and length of hospitalizations, death rates, and medication.

### Dual-task assessment

The secondary tasks included in the dual-task evaluation will be of three different natures: 1) semantic fluency: naming animals or fruits and vegetables; 2) backward counting by ones; and 3) inhibition ability through the Go no go test (when the evaluator says ‘one’, the participant has to respond ‘two’ and viceversa). We selected these tasks by the following process: a) a review of the literature, b) expert consultation through interviews and a discussion group, c) final selection.

Cognitive tasks will be applied during two different physical function tests: 9 m usual gait speed and the Timed Up and Go test. In addition, these physical tasks will be performed in a single task mode to allow for dual-task cost calculation. Dual-task gait speed and dual-task Timed Up and Go tests will be performed on two non-consecutive testing days to minimize learning effects and the order of the dual-task and the single-task will be randomized for the same purpose. In addition, participants will wear an accelerometer (BTS Biomedical G-WALK triaxial accelerometer and gyroscope) during the tests to measure gait kinematic parameters such as step number, cadence, step symmetry, and step time variability. The number of total responses, errors, repetitions, and stops will be recorded. No instructions will be given regarding task prioritization.

### Safety assessments

All co-existing diseases or conditions related to the intervention will be treated in accordance with prevailing medical practice and will be reported as an adverse event. In cases where the functional and cognitive state of a participant decreases due to an adverse event (e.g. illness, falls, etc.) the program will be individualized and adapted for that person upon her/his return.

### Power and sample size

Sample size for the current study was calculated to detect a significant clinical difference on the dual-task gait speed test [54]. Accepting an alpha risk of 0.05 and a beta risk of 0.20 in a bilateral contrast, 141 individuals are required to detect a difference equal to or greater than 0.08 m/seg in the dual-task gait speed test (SD = 0.24). The sample size was increased by 20% to account for losses during follow-up and an additional 5% for mortality. The resulting sample size is 188 individuals, allocating 94 participants to each group.

### Statistical considerations

Data analysis will be performed using the IBM SPSS Statistics 24 statistical software package (SPSS, Inc., Chicago, IL). Intention-to-treat analyses will be performed and the level of statistical significance will be set at *p* < .05 for all computations. First, all data will be checked for normality of distribution using the Kolmogorov-Smirnov test. Results will be expressed as mean (with standard deviation) for continuous and normally distributed variables and as median (with interquartile range) when normality of data for that variable cannot be assumed. In the case of categorical variables, frequency counts and percentages will be used to describe the results. Tests for baseline comparisons will be selected based on the nature and distribution of the data: Student’s-t test with continuous and normally distributed variables, Mann-Whitney test with non-normally distributed continuous variables, and Chi-squared test with categorical variables.

To test the effects of training interventions, mixed-designed ANCOVA-s or the Friedman test, including baseline measurements, age, or gender as covariates, will be performed for physical, cognitive, and emotional variables. In cases where a significant F value is found, LSD post hoc procedures will be performed for pairwise comparisons.

## Discussion

The current trial is a large multi-center randomized study aiming to investigate whether dual-task performance, including gait and cognitive parameters, can be improved by specific dual-task training. So far, guidelines for the geriatric population and professionals working in the field are scarce, despite the exponentially increasing number of people above 65 years old. Older adults in long-term nursing homes are at particular risk of adverse outcomes and have been the focus of interventions aiming to prevent or reverse frailty [55].

The results of the present study will add valuable knowledge about the effects of the dual-task program in long-term nursing home residents, taking together functional, cognitive, and emotional variables linked to frailty. Particularly, analysis of a multicomponent exercise program and the same program with simultaneous cognitive training, or dual-task, will help us to design interventions to improve or at least maintain functionality and cognition in long-term nursing home residents.

One of our main concerns at the time of designing the dual-task intervention was the fact that when performing a dual-task exercise, the execution velocity of the physical task could be reduced when compared with a single task exercise. In addition, movement technique could also be altered if compared to single task training. Consequently, we feared that a dual-task program might affect physical performance and hence not improve physical parameters to the same extent as the multicomponent program. Thus, we conducted a pilot study to ascertain if both the multicomponent and the dual-task programs produce similar training adaptations, in which we successfully observed significant physical improvements in both groups [56].

Methodological strengths of the present study include the fact that the dual-task program here is based on a previously published physical exercise protocol. This protocol was feasible and demonstrated improvements in many functional outcomes [32, 33]. In addition, the proposed interventions are easy to deliver and include exhaustive practical information regarding implementation such as training frequency, volume, intensity, individualization, and resting periods. This will allow an easy and straightforward implementation in long-term nursing homes. The existing literature about exercise protocols for older adults living in long-term care facilities includes few randomized controlled trials and the methodology tends to be heterogeneous. Furthermore, description of the methods used is often not enough to allow for replication.

We also recognize possible limitations to the study. The selected inclusion criteria preclude the majority of long-term nursing home residents, as we will include light to moderately dependent subjects while the prevalent profile in this type of institution is severely dependent. Consequently, we might encounter difficulties reaching the desired sample size. However, the large number of agreements made with long-term care institutions will facilitate the recruitment of enough subjects.

The proposed interventions will help to define the best approach to prevent the functional, cognitive, and emotional decline associated with age in older adults living in long-term nursing homes, considering feasibility and adherence.

## Abbreviations

I.A.N.A: International Academy on Nutrition and Aging
iTUG: instrumented Timed Up and Go test
MEC-35: Mini Examen Cognoscitivo
MMSE: Mini Mental State Examination
MoCA: Montreal Cognitive Assessment
QoL-AD: Quality of Life Alzheimer’s disease scale
RAVLT: Rey Auditory Verbal Learning Test
SFT: Senior Fitness Test
SPPB: Short Physical Performance Battery test
TMT: Trail Making Test
WAIS-IV: Wechsler Adult Intelligence Scale, Fourth Edition
